# Electroglottography of speakers of Brazilian Portuguese through Objective Multiparameter Vocal Assessment (EVA)

**DOI:** 10.1590/S1808-86942012000400007

**Published:** 2015-10-20

**Authors:** Bárbara Silveira de Faria, Karina Vitor de Oliveira, Juliana Preisser Godoy e Silva, César Reis, Alain Ghio, Ana Cristina Côrtes Gama

**Affiliations:** Speech and Hearing Therapist (Speech and Hearing Therapist); MSc in Linguistics (Speech and Hearing Therapist); PhD in Linguistics (Associate Professor at the Literature and Languages School of the Federal University of Minas Gerais); PhD in Sciences (Research Engineer); PhD in Voice Disorders (Associate Professor at the Department of Speech and Hearing Therapy of the Federal University of Minas Gerais). Faculdade de Letras e Faculdade de Medicina da Universidade Federal de Minas Gerais

**Keywords:** speech acoustics, speech therapy, voice

## Abstract

EVA was designed to study various speech production parameters.

**Objective**: This paper aims to define the mean values for electroglottography tests of Brazilian Portuguese speakers on EVA.

**Materials and Method**: The voices of 20 men and 20 women without voice-related complaints were analyzed through electroglottography so as to obtain reference values for normality. Case study: this is a descriptive cross-sectional study.

**Results**: The mean values for normal male voices were: F0 = 127.77 Hz; F0 coefficient of variation = 2.51%; *absolute jitter* = 1.707 Hz; relative average perturbation = 0.0083; *jitter factor* = 1.34%; *jitter ratio* = 13.45%; QF = 0.447. The values for female voices were: F0 = 204.87 Hz; F0 coefficient of variation = 1.58%; *absolute jitter* = 3.30Hz; relative average perturbation = 0.0102; *jitter factor* = 1.60%; *jitter ratio* = 16.23%; QF = 0.443. Wave type for the entire sample was categorized as tilted pulse.

**Conclusion**: Statistically significant differences were found for gender on parameters average F0 and *absolute jitter*. While using acoustic analysis software, users must be based on parameters inherent to the software program when analyzing the collected data.

## INTRODUCTION

Voice assessment in speech therapy can be performed through auditory perceptual analysis, considered the gold standard in speech therapy, or through acoustic analysis, a set of measurements performed from computer-generated tracings^1^.

Acoustic analysis added objectivity to speech assessment. Additionally, it allowed increased diagnostic accuracy, the identification and documentation of short and long term therapy results, and the possibility of providing patients with visual feedback^2^.

Electroglottography (EGG) is a non-invasive test that estimates the contact area variation between vocal folds as voice is produced^3^. It has been used in acoustic analysis since the 1940s in clinical and research settings^4^.

A center in France dedicated to studying speech and language developed a multiparameter method for objective assisted voice assessment (EVA) that uses the SESANE data processor. EVA was designed to study parameters in speech production such as sound, intensity, aerodynamic measurements, to name a few. It is equipped with a series of sensors to measure these parameters, and thus offers improved diagnostic capabilities and enhanced patient follow-up in terms of surgery, drug therapy, and phototherapy outcome^5^.

Acoustic analysis software for speech and voice differ in the way they calculate acoustic parameters, and the outcome of the measurements may be affected by linguistic variations stemming from language cultural patterns^6^. Results also vary depending on the recording instrumentation, ambient noise, gender or age of the speaker, which shows that the quality of the equipment used to record patient voice, the type of software, and the anatomical-functional of the larynx may affect measurements in the short run^1^. Normative values can only be assessed by means of standardized criteria agreed upon by consensus^7^. Standardization educates, simplifies, saves time, money and effort, aside from ensuring certification^8^.

There are no studies in the literature describing the use of EVA-based electroglottography in Brazilian Portuguese speakers.

The purpose of this study is to analyze mean values for fundamental frequency (F0), F0 coefficient of variation, *absolute jitter*, relative average perturbation (*RAP), jitter ratio, jitter factor*, mean closed quotient (CQ), and the interpretation of the electroglottography wave types of the EGG/EVA software, so as to gather preliminary data on normal patterns of speakers of Brazilian Portuguese of both genders.

## MATERIALS AND METHODS

This is a descriptive cross-sectional study. Forty native speakers of Brazilian Portuguese - 20 males and 20 females - aged between 18 and 45 years were enrolled. The selected age range aimed at excluding individuals experiencing changes in their voices and presbyphonia. The mean age of female subjects was 28 years; male patients had a mean age of 30 years.

None of the subjects had voice-related complaints. Auditory perceptual analysis performed by two speech and hearing therapists did not show altered voice quality or any other communication disorder that could prevent them from performing the tests.

Enrolled subjects were informed of the purpose, procedures, and publication of the test results in this study, and signed an informed consent form. This study was approved by the Research Ethics Committee of our institution and was granted permit ETIC 0488.0.203.000-10.

All subjects had their voices recorded in acoustic signals and electroglottography and were asked to say the phrase *“Mara lava a batata”* twice in a row. The second utterance of the phrase was used for data analysis purposes due to its increased acoustic stability and the utterance of vowel/a/in syllable/la/, as it is assumed that there is lesser influence from the vocal tract, given that the tonic syllable is located in the more central portion of the phrase.

Acoustic analysis software EVA was used to record and analyze speech samples. Recordings were done using a Dell Vostro 200 workstation and a professional -44 dBV AKG Acoustics C1000S condenser stereo multidirectional microphone. Two electrodes were placed on the wings of the thyroid cartilage and the informant was kept at a fixed 10 cm from the microphone to allow for proper capturing of the electroglottography signal.

Electroglottography measurements were made so as to obtain reference values for mean fundamental frequency (F0), F0 coefficient of variation, *absolute jitter*, relative average perturbation (*RAP*), *jitter ratio*, *jitter factor*, and closed quotient (CQ) (Table 1). The software program's manual contains a detailed description of all analyzed parameters defined below^9^.

**Table 1:** Normality reference values for EGG/EVA defined on the software program's manual.

Parameters	Minimum	Maximum
*Absolute Jitter (Hz)*	0.3	4
*RAP*	0.003	0.01
*Jitter Factor (%)*	0.99	5
*Jitter Ratio (%)*	8	15
F0 coefficient of variation (%)	1.5	4

*RAP*: Relative average perturbation.

Mean F0 offers a general measurement of vocal frequency which corresponds to the number of sound waves comprised within one second. The unit of measurement is Hertz (Hz).

F0 coefficient of variation is the relative standard deviation compared against the mean F0. This measurement accounts for the magnitude of percent changes in comparison to the mean F0 value. For example, a standard deviation of 4.9 Hz for a mean F0 of 180 Hz results in a coefficient of variation of 2.7%. The same standard deviation for a mean F0 of 500 Hz provides for a much more significant coefficient of variation of 0.98%. The F0 coefficient of variation is the best indicator to explore the stability of the mean fundamental frequency duration, and is highly relevant in the detection of alterations such as tremor and other instabilities of neurological origin. It is measured as a percentage (%).

Short term instability (*absolute jitter)* of the F0 results shows the changes in frequency between each oscillation cycle. It is calculated using *absolute mean jitter* and the mean F0 difference between two consecutive vibration cycles. These alterations can be accurately calculated for each cycle. It is measured in Hertz (Hz).

Relative average perturbation (*RAP)* measures the mean variation in three consecutive periods and denotes the mean period of the observed signal. This variable has no unit of measurement.

*Jitter factor* establishes a ratio between mean *absolute jitter* and mean F0. A *mean jitter* of 0.677 Hz and a mean F0 of 180 Hz correspond to a *jitter factor* of 0.38%. *Jitter factor* is a great indicator to explore the short term stability of the fundamental frequency. It is measured as a percentage (%).

*Jitter ratio* measures the mean variation seen in a period between two consecutive vibration cycles. A high *jitter ratio* always signifies a relevant F0 coefficient of variation, although the opposite is not true. Indeed, the small upward or downward variations on F0 between cycles does not produce a relevant *jitter ratio*, but may lead to significant global F0 variations, such as vibrato. The unit of measurement is permillage (%).

**Figure 1:**
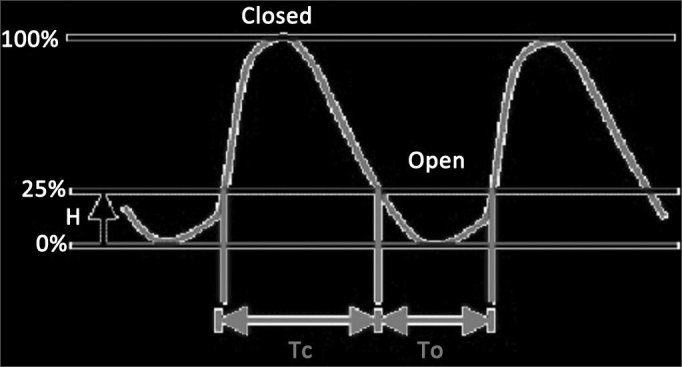
Diagram to visualize electroglottographic waves and calculate closed quotient. Time closed (Tc), complete glottal cycle (Tc + To), limit amplitude (H). Source: http://www.sqlab.fr/

The closed quotient measures the ratio between the time closed (Tc) and the complete glottal cycle (Tc + To): CQ = Tc/(Tc + To). It is expressed as a percentage (%) (Figure 1).

The EVA software manual states that CQ normal values, based on French speakers, range between 0.4 and 0.6. Values between 0 and 0.4 suggest glottal hypoadduction and values greater than 0.6 and smaller than 1.0 suggest glottal hyperadduction.

**Figure 2:**
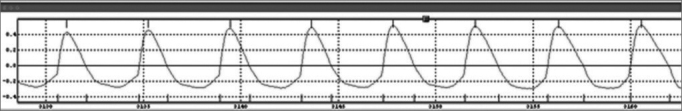
Model of an electroglottographic wave obtained through EGG/EVA recording. Electroglottographic signal Amplitude vs. Time. Source: EVA Manual.

In addition to closed quotient, electroglottographic waves were qualitatively analyzed, categorized, interpreted according to waveform characteristics, and related to templates of glottal geometric variation^10^:

1. Pulse widening: occurs when the free border shifts uniformly towards the midline;
2. Peak skewing: occurs when there is increased glottal convergence, i.e., when a vocal fold is more acutely angled and wedged;
3. Bulging pulse: occurs when two knees are seen in the tracing, one going up and another going down;
4. Sloping pulse: occurs when there is a slight difference in the phase angles between and upper and lower margins of the vocal fold free borders, changing the waveform to a more quadrangular or triangular shape when the angle difference between upper and lower margins is greater^11^ (Figure 2).

Data statistical analysis was carried out using statistical package SPSS (Statistical Package for the Social Sciences) release 17.0. Initially, a descriptive analysis of the data was performed looking at central tendency and scatter measurements. The data followed a normal distribution. Therefore, the statistical analysis of the values between genders was done using Student's t-test with a confidence level of 95%.

## RESULTS

Table 2 shows minimum and maximum values, standard deviation and level of significance of electroglottographic measurements in females and male individuals.

**Table 2:** Minimum and maximum values, standard deviation, mean values, and p-values of electroglottographic parameters of male and female subjects.

	Gender	Minimum	Maximum	SD	Mean	*P*
Mean F0	Female	168.27	270.80	25.62	204.87	0.000
	Male	99.69	174.92	19.89	127.77	
F0 coefficient of variation	Female	0.90	2.62	0.52	1.58	0.078
	Male	0.58	8.60	2.24	2.51	
*Absolute jitter*	Female	1.33	6.36	1.37	3.30	0.000
	Male	0.61	4.37	1.02	1.70	
*RAP*	Female	0.00	0.03	0.01	0.01	0.049
	Male	0.00	0.04	0.01	0.01	
*Jitter factor*	Female	0.09	3.36	0.81	1.60	0.285
	Male	0.38	2.63	0.72	1.34	
*Jitter ratio*	Female	6.55	36.27	7.68	16.23	0.247
	Male	3.81	26.43	7.29	13.45	
Closed quotient	Female	0.327	0.54	0.063	0.443	0.835
	Male	0.341	0.499	0.05	0.447	

*p*-value < 0.05. Student's *t*-test. F0: Fundamental frequency; *RAP*: Relative average perturbation; SD: Standard-deviation.

There is statistically significant difference between genders for mean F0 and *absolute jitter* measurements.

In the analysis of electroglottographic wave type according to Titze^10^, for both studied groups 100% of the subjects had *peak skewing* wave types.

## DISCUSSION

Electroglottography looks into the contact pattern of vocal folds during the glottal cycle to assess vocal function^12^. A high frequency low amplitude charge is applied to the subject's neck structures and vocal folds through electrodes placed bilaterally on the neck^13^.

Human tissues conduct electricity reasonably well when compared to air^14^. The opening and closing of vocal folds cause impedance levels to vary in the larynx, thus altering the flow of electricity between the electrodes^12^. Current levels are affected by resistance levels, and consequently by tissue impedance^15^.

When vocal folds touch there is some flow of electricity, and as they move away from each other flow is significantly reduced. Only a small portion of the flow of electricity recorded shows the contact between vocal folds^16^.

The resulting electroglottogram (EGG) shows the variation of vocal fold impedance as a function of time. Impedance also varies considerably with skin type and vertical laryngeal motion. High-pass filters are used to eliminate low frequency interference and remove the variation caused by vocal fold vibration^17^.

Various objective measurements may be gathered from the analysis of electroglottograms. Parameters such as vibration fundamental frequency, amplitude perturbation, *shimmer*, frequency perturbation, *jitter*, and closed quotient^18^.

Altuzarra & San Martin^19^ reported that EGG is a broadly accepted method to measure fundamental frequency and F0 perturbation.

Electroglottogram tracings can be interpreted in many different ways. One may consider the configuration of the tracing curves, their amplitude, cycle periodicity, and the presence or absence of knees^10^. The electroglottogram waveform reflects the amount of cross-sectional impedance at the level of the larynx; impedance readings fall as vocal fold contact increases^20^. Vocal function can be assessed by measuring the variations in contact time of the vocal fold mucosa in the posteroanterior and inferosuperior direction of the free border during a vibration cycle^10^.

Electroglottographic studies^11^ performed in female patients without functional or anatomical disorders of the vocal tract showed a mean F0 value of 211.69 Hz with a standard deviation of 15.13, and a mean closed quotient of 0.455 with a standard deviation of 0.033. These results support this study (Table 2) in terms of mean F0 (204.87 Hz) and closed quotient (0.443) values. Nevertheless, the standard deviations found in this study were higher than those presented in the paper mentioned above. These differences may be explained by the fact that the measurements were captured using different electroglottographic systems.

The control group of a study^21^ looking into EGG findings in individuals with multiple sclerosis found mean F0 values and *jitter factors* similar to those reported in this study (Table 2), probably due to the similarities in subject age range and research method.

A study^22^ that analyzed EGG findings of laryngeal tumor patients reported a mean F0 of 133.80 Hz and a mean *jitter factor* of 0.23% among members of the control group. The values found in our study were different from those cited above. The study mentioned above did not analyze their groups for gender, therefore data from male and female patients were combined. This difference may have contributed for the marked differences seen between studies, although the findings reported in the study mentioned above are similar to the data for the male subjects enrolled in our study.

Fundamental frequency is more easily derived from electroglottograms than from sound wave acoustic analysis as cycles can be seen more clearly, thus confirming the increased reliability in obtaining F0 data from EGG^14^, ^19^. The mean F0 and standard deviation values seen in two studies^23^, ^24^ that analyzed Portuguese and brazilian portuguese speakers found practically identical values as those reported in this study (Table 2).

There was a match in closed quotient values for male (CQ = 0.447) and female (CQ = 0.443) subjects enrolled in this study (Table 2) when compared to other studies^11^, ^24^, ^25^, ^26^, thus confirming that individuals without laryngeal disorders, mainly nodules^27^, have CQ within normal ranges.

It is worthwhile mentioning that the statistically significant difference observed between male and female groups (Table 2) for mean F0 was also reported in other studies^20^. There are no other papers in the literature reporting on other EGG parameters having gender as a reference.

The electroglottograms of all patients enrolled in this study had skewing peaks according to the categorization proposed by Titze^10^, as also reported in other studies^11^, ^28^. Peak skewing occurs when there is increased glottal convergence, in situations where the vocal folds do not have free border disorders and show adequate closing^29^.

The parameters considered to assess normality among French speakers are F0 coefficient of variation, *absolute jitter*, relative average perturbation (*RAP), jitter ratio*, and *jitter factor*.

The values mentioned above (Table 1) do not match the findings reported in this study (Table 2). Our results relate to Brazilian Portuguese speakers, and the linguistic variations associated with the language's cultural standards may also affect speech and voice patterns. Those factors combined may lead to significant differences in the acoustic electroglottographic findings of speakers of different languages^6^.

More electroglottographic research using different software programs and looking into other languages is needed to allow for a better understanding of these variables and, consequently, to improve the analyses of these values in subjects with speech and laryngeal disorders. Utter standardization is not possible, as there will always be differences between software programs for speech acoustic analysis. Therefore, when using a software pro gram for acoustic analysis, users must use as reference the parameters inherent to the program they are using to analyze the collected data samples.

## CONCLUSION

The mean reference values for normality found in this study for Brazilian Portuguese speakers without voice- related complaints were: male subjects - F0 = 127.77 Hz; F0 coefficient of variation = 2.51%; *absolute jitter* = 1.707 Hz; relative average perturbation (*RAP)* = 0.0083; *jitter factor*= 1.34%; *jitter ratio* = 13.45%; closed quotient (CQ) = 0.447. Female subjects - F0 = 204.87 Hz; F0 coefficient of variation = 1.58%; *absolute jitter* = 3.30 Hz; relative average perturbation (*RAP)* = 0.0102; *jitter factor* = 1.60%; *jitter ratio* = 16.23%; closed quotient (CQ) = 0.443.

The electroglottographic parameters that presented gender statistically significant differences were mean F0 and *absolute jitter*.

*Peak skewing* waveform was found in the electroglottograms of 100% of the subject sample of both genders.

## References

[1] Valentim AF, Côrtes MG, Gama ACC. (2010). Análise espectrográfica da voz: efeito do treinamento visual na confiabilidade da avaliaçã o. Rev Soc Bras Fonoaudiol.

[2] Teles VC, Rosinha ACU. (2008). Análise acústica dos formantes e das medidas de perturbaçã o do sinal sonoro em mulheres sem queixas vocais, não fumantes e não etilista. Arq Int Otorrinolaringol.

[3] Colton RH, Conture EG. (1990). Problems and pitfalls of electroglottography. J Voice.

[4] Fabre P. (1940). Sphygmographie par simple contact délectrodes cutanes, introduisant dans l arterè de faibles courants de haute fréquence détecteurs de ses variations volumétriques. Comptes Rendus Soc Biol.

[5] SQLab (2011). Disponível em: Acesso em: 14 de junho de.

[6] Felippe ACN, Grillo MHMM, Grechi TH. (2006). Standardization of acoustic measures for normal voice patterns. Braz J Otorhinolaryngol.

[7] Kania RE, Hans S, Hartl DM, Clement P, Crevier-Buchman L, Brasnu DF. (2004). Variability of electroglottographic glottal closed quotients: necessity of standardization to obtain normative values. Arch Otolaryngol Head Nech Surg.

[8] Titze IR., The G. (1994). Paul Moore Lecture. Toward standards in acoustic analysis of voice. J Voice.

[9] Ghio A. (2008). Laboratoire Parole et Langage Universite de Aix-en-Provence.

[10] Titze IR. (1990). Interpretation of the electroglottographic signal. J Voice.

[11] Mourão AM, Bassi IB, Gama ACC. (2011). Avaliaçã o Eletroglotográfica de mulheres disfônicas com lesão de massa. Rev CEFAC.

[12] Herbst CT, Howard D, Schlömicher-Thier J. (2010). Using electroglottographic real-time feedback to control posterior glottal adduction during phonation. J Voice.

[13] Horiguchi S, Haji T, Baer T, Gould WJ. (1991). Comparison of eletroglorro-graphic and acoustic waveform perturbation measures.

[14] Behlau M, Madazio G, Pontes P., Behlau M (2001). Voz: o livro do especialista.

[15] Avelino H. (2010). Acoustic and electroglottographic analyses of nonpathological, nonmodal phonation. J Voice.

[16] Colton RH, Woo P. (1995). Treatment of Voice Disorders.

[17] Mattos JS, Silva DG, Junior JAA, Cataldo E. (2008). Incursionando pelos domínios da eletroglotografia: proposta de um corpus EGG.

[18] Rothenberg M. (1992). A multichannel electroglottograph. J Voice.

[19] Altuzarra NA, San Martin RE. (1996). Electoglotografia. Diagnóstico y tratamiento de los transtornos de la voz.

[20] Ma EP, Love AL. (2010). Electroglottographic evaluation of age and gender effects during sustained phonation and connected speech. J Voice.

[21] Konstantopoulos K, Vikelis M, Seikel JA, Mitsikostas DD. (2010). The existence of phonatory instability in multiple sclerosis: an acoustic and electroglottographic study. Neurol Sci.

[22] Kazi R, Venkitaraman R, Johnson C, Prasad V, Clarke P, Newbold K (2008). Prospective, longitudinal electroglottographic study of voice recovery following accelerated hypofractionated radiotherapy for T1/T2 larynx cancer. Radiother Oncol.

[23] Guimarães I, Abberton E. (2005). Fundamental frequency in speakers of Portuguese for different voice samples. J Voice.

[24] Silva VOS. (1999). Análise eletroglotográfica de diferentes tipos de vozes. [trabalho de conclusão de curso].

[25] Chen Y, Robb MP, Gilbert HR. (2002). Electroglottographic evaluation of gender and vowel effects during modal and vocal fry phonation. J Speech Lang Hear Res.

[26] Lim JY, Lim SE, Choi SH, Kim JH, Kim KM, Choi HS. (2007). Clinical characteristics and voice analysis of patients with mutational dysphonia: clinical significance of diplophonia and closed quotients. J Voice.

[27] Hall KD. (1995). Variations across time in acoustic and electroglottographic measures of phonatory function in women with and without vocal nodules. J Speech Hear Res.

[28] Bogossian CB. (1998). Análise Eletroglotográfica em mulheres adultas disfôni-cas com nódulos vocais. [trabalho de conclusão de curso].

[29] Childers DG, Hicks DM, Moore GP, Alsaka YA. (1986). A model for vocal fold vibratory motion, contact area, and the electroglottogram. J Acoust Soc Am.

